# Safe and soothed: randomised clinical pilot study on the subjective and psychophysiological impact of a new physiotherapeutic intervention in patients with dissociative disorders

**DOI:** 10.1192/bjo.2025.10072

**Published:** 2025-09-10

**Authors:** Lea Stief, Heribert Sattel, Karin Paschinger, Martin Sack, Eva Schäflein

**Affiliations:** Department of Psychosomatic Medicine and Psychotherapy, University Hospital Rechts der Isar Munich, Technical University of Munich (TUM), Germany; Department of Orthopaedics and Sports Orthopaedics, Physical Therapy, University Hospital Rechts der Isar Munich, Technical University of Munich (TUM), Germany; Department of Psychotherapy and Psychosomatic Medicine, Faculty of Medicine, Technische Universität Dresden (TUD), Germany; Department of Psychosomatic Medicine and Psychotherapy, University Hospital Erlangen, Friedrich-Alexander-University Erlangen-Nuremberg (FAU), Germany

**Keywords:** Dissociative disorders, post-traumatic stress disorder, physiotherapy, psychophysiology, safety

## Abstract

**Background:**

Dissociative disorders frequently co-occur with post-traumatic stress disorder (PTSD), yet many individuals lack adequate treatment. Existing interventions often prioritise reducing arousal over promoting safety and self-soothing, tending to neglect the bodily experience.

**Aims:**

This randomised clinical within-person pilot study examined the effects of the nest position, a physiotherapeutic intervention designed to enhance safety and self-soothing, on patients with dissociative disorders and healthy controls (German Clinical Trials Register No.: DRKS00030669).

**Method:**

Eighteen patients with dissociative disorders and 18 healthy controls alternated between the nest position and a neutral supine position across two rounds of a measurement session. The order of the experimental conditions (nest position or supine only) was randomised for each participant. We assessed self-reported distress and comfort (Subjective Units of Distress and Comfort) and autonomic nervous system activity during three baseline phases and imagination of stressful and comforting situations.

**Results:**

Both patients and healthy controls experienced lower distress and greater comfort in the nest position. Heart rate and sympathetic tone decreased, particularly in the healthy controls. There were no significant changes in parasympathetic tone in both groups. Linear mixed models revealed a significant effect of the nest position on distress, comfort and sympathetic tone.

**Conclusions:**

The nest position is a potentially promising additional intervention for highly dissociative patients. Our findings help to better understand the importance of self-soothing and safety in these individuals and to address the research gap in physiotherapy within in-patient mental health care.

According to the ICD-11 and the DSM-5-TR, dissociative disorders are characterised by a disruption in the normal integration of psychological and somatic functions, e.g. memory, sense of identity and the perception and control of bodily movements.^1,2^ Despite the prevailing belief that dissociative disorders are rare, epidemiological studies indicate a prevalence of approximately 10% in psychiatric settings, with ‘Dissociative Disorders Not Otherwise Specified’ (DDNOS) being the most common diagnosis.^3^ DDNOS are characterised by various symptoms that do not fully meet the criteria for other defined dissociative disorders. In the current DSM-version, this term has been updated to Other Specified Dissociative Disorders (OSDD), which encompasses most cases previously classified as DDNOS.^2^

Dissociative symptoms are a transdiagnostic phenomenon prevalent in numerous mental disorders, including post-traumatic stress disorder (PTSD), borderline personality disorder, schizophrenia and anxiety disorders.^4^ In individuals with PTSD, not only detachment symptoms such as depersonalisation and derealisation may occur, but also more severe dissociative symptoms, e.g. identity disturbance and amnesia.^5^

However, many highly dissociative individuals do not receive adequate treatment: in a study by Nester et al,^6^ 96.7% of participants reported significant difficulties in finding treatment tailored to their specific needs. Given that dissociative disorders are associated with an increased risk of self-harm and suicidality^7^ as well as poor treatment response,^8^ it is imperative to develop new therapeutic interventions that can augment existing effective treatment regimens, ideally within a multimodal treatment framework.

## Current treatment of dissociative disorders

Presently, there is only one consensus-based guideline on the treatment of dissociative disorders that outlines a three-phase approach to treatment: stabilisation and symptom reduction, confronting and processing traumatic memories, and achieving personality integration and rehabilitation.^9^ According to the guideline, effective treatment employs a multimodal framework to address the patients’ various challenges and aids in developing safety and stability in everyday life.^9^

To effectively manage negative emotions and psychophysiological arousal without resorting to self-injurious behaviour, establishing fundamental self-regulation strategies in the initial phase of treatment is crucial. Consequently, two key objectives in this stage are to foster adequate or improved affect regulation and provide safety. This aims to increase the capacity to attain states of comfort and calm rather than emotional distress and to build stress tolerance. Until a foundation of safety is established, other treatment issues may need to be postponed.^9^

Multimodal treatment approaches that involve the body, e.g. by integrating physiotherapeutic elements, are gaining increasing attention due to their potential benefits for patients in safely expressing their affective states non-verbally.^9^ They not only facilitate the communication of vital information, such as triggers or safety concerns, before patients are able to verbalise them, but also aid in balancing dysregulated physical states that contribute to dissociation.^9^ However, such approaches remain inadequately researched and lack sufficient scientific evidence. To our knowledge, the available studies in the field of dissociative disorders concerning the use of physiotherapy have exclusively examined functional neurological disorders/conversion disorders (e.g. ^10^).

## Self-soothing and safety in dissociative disorders

As mentioned earlier, effective treatment interventions should focus on promoting feelings of safety and facilitating emotion regulation as well as self-soothing to reduce the patients’ subjective sense of being at the mercy of their involuntary behaviour. These aspects also play a crucial role in the aetiology of the disorder. For instance, a lack of safety and comforting experiences during childhood is linked to more pronounced disorganised attachment in infants, which, in turn, is a key precursor of dissociation.^11^ Conversely, childhood experiences of safety serve as a protective factor against dissociation.^12^ As Barlow et al^13^ report, difficulties in emotion regulation account for the relationship between childhood abuse and symptoms of PTSD.

The ability to self-soothe is not innate but, rather, acquired through positive relationships with caregivers during childhood. In their first months of life, infants rely on others to regulate their internal state, especially in emotionally overwhelming situations.^14^ Similarly, the experience of safety is a component of affect regulation; more specifically, it is a distinct dimension of positive affect. Gilbert et al^15^ propose the existence of a discrete emotion regulation system dedicated to self-soothing and seeking safeness. Furthermore, the Generalized Unsafety Theory of Stress posits that not perceiving a situation as safe is linked to the development of (chronic) stress.^16^ According to this theory, the stress response is automatically activated unless clear signals of safety are present. Even in situations that, on their own, do not elicit distress, the environment is perceived as generally unsafe.

## The present study

**Fig. 1:**
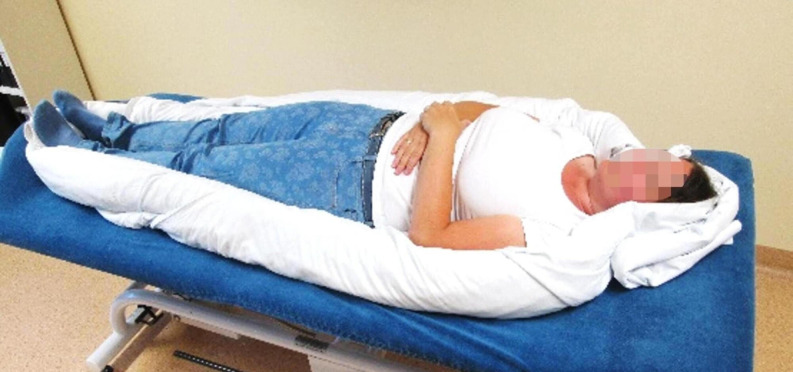
The nest position in supine position. The patient is surrounded by two blankets which have been rolled tightly from one corner to the other. The head and the soles of the feet are surrounded with small rolled towels or additional blankets. If necessary, towels can also be pushed under the blankets at the sides of the legs to prevent the blankets from coming loose. Other material such as special positioning rolls of different lengths and stuffing can be used. The patient should feel the material touching all parts of their outer body borders. The nest position can also be adjusted individually in the sitting or lateral position. (© K. Paschinger, with the patient’s consent.)

Although the crucial role of interventions addressing safety and self-soothing in dissociative disorders has been recognised by researchers and is included in the current treatment guideline, little is known regarding their effectiveness and efficacy. Thus, the aim of this pilot study was to investigate such an intervention that promotes safety and facilitates self-soothing. We are the first to employ a physiotherapeutic intervention, known as the nest position, in adult patients with dissociative disorders. The concept was developed by co-author and physiotherapist K.P. and has so far not been utilised in the treatment of trauma-related disorders. Patients are surrounded or cocooned by two blankets and other soft materials, such as pillows and towels, while lying on their back (Fig. 1). To minimise invasiveness, this involves only handling the soft material when positioning it around the body, rather than direct physical contact with the patient. The name reflects the form of this position and symbolises the state of safety and comfort the bedding is intended to evoke.

In the present pilot study, we assessed the subjective and psychophysiological experience of stress and comfort during the nest position compared to a neutral supine position. We hypothesised that the nest position would significantly reduce self-reported distress and increase self-reported comfort during three baseline phases as well as during imagination of a stressful situation, as compared to a neutral supine position. We further hypothesised that – similarly – the nest position would significantly increase the parasympathetic drive and reduce the sympathetic drive, both during all three baseline phases as well as during imagination of a stressful situation. We expected these effects in adult patients with dissociative disorders as well as in healthy controls.

## Method

This randomised clinical within-person pilot trial was retrospectively registered at the German Clinical Trials Register (DRKS00030669). The authors assert that all procedures contributing to this work comply with the ethical standards of the relevant national and institutional committees on human experimentation and with the Helsinki Declaration of 1975, as revised in 2013. All procedures involving human subjects/patients were approved by the ethics committees of the Technical University of Munich (338/17 S; 03/08/2017) and the Friedrich-Alexander University Erlangen-Nuremberg (411_20 Bc; 16/10/2020).

In this exploratory pilot study, the sample size was calculated to detect large effect sizes (*d* = 0.8) in intra-group comparisons with two-tailed *t*-tests, assuming a significance level of *α* = 0.05 and a statistical power of 80%. Based on these parameters, a sample size of 15 patients and 15 healthy controls was deemed appropriate (calculated by G*Power for Windows, version 3.1.9.4, University Düsseldorf, Düsseldorf, Germany; see https://www.gpower.hhu.de). To account for an estimated 20% drop-out rate, we aimed to recruit 18 participants per group.

### Participant selection

Between 21 May 2019 and 15 December 2020, we recruited 18 female in- and out-patients from the Departments of Psychosomatic Medicine and Psychotherapy at the University Hospital Rechts der Isar Munich, Germany (*n* = 11), and the University Hospital Erlangen, Germany (*n* = 7). Additionally, 18 healthy controls – employees and students from the University Hospital Rechts der Isar Munich as well as healthy participants from previous studies who had agreed to be recontacted – were recruited and matched to the patients by gender, body mass index (BMI) and age (5 June 2019 to 18 October 2021). By comparing highly traumatised, dissociative patients with healthy controls, we aimed to identify the specific effects of the nest position on individuals with potential deficits in experiencing safety and self-soothing.

To be eligible for this study, patients required a diagnosis of dissociative disorder, specifically DDNOS Type 1 according to the DSM-IV, and PTSD, both confirmed by structured clinical interviews. DDNOS Type 1 is a subthreshold form of dissociative identity disorder in which all of the diagnostic criteria of dissociative identity disorder except for amnesia or identity alteration are present.^2^ Patients also exhibited other psychiatric comorbidities, e. g. affective or eating disorders, while healthy controls had to be free of mental illnesses. The exclusion criteria for both groups included psychotic and substance abuse disorders, severe physical diseases and the use of benzodiazepines, beta-blockers or antiarrhythmic drugs.

During an initial interview, participants were informed about the study procedure and potential adverse effects. We did not disclose details about the nest position, especially its name and method, describing it only as an innovative physiotherapeutic intervention. The exact measurement phases and hypotheses were also withheld to minimise bias. Written informed consent was obtained and an appointment for the measurement session was scheduled for eligible participants. They also received paper-and-pencil self-report questionnaires to complete before the experiment.

### Diagnostic interviews and self-report questionnaires

We used the short form of the Structured Clinical Interview for DSM-IV Dissociative Disorders (Mini-SCID-D) to diagnose DDNOS.^17,18^ It comprises the subscales amnesia, depersonalisation, derealisation, identity confusion and identity alteration. To be included in the study, patients needed to achieve a total score of ten or higher (range: 0–15). PTSD was diagnosed using the PTSD module of the Structured Clinical Interview for DSM-IV (SCID-PTSD).^19^

We measured child abuse and neglect with the Childhood Trauma Questionnaire (CTQ), a screening tool with five subscales (emotional abuse, physical abuse, sexual abuse, emotional neglect, physical neglect).^20^ To evaluate trait dissociation, we administered the Dissociative Experience Scale (DES).^21^ General psychological distress was measured using the Brief Symptom Inventory (BSI), which is the short form of the revised Symptom Checklist-90 (SCL-90-R).^22,23^ Intrusion and avoidance related to PTSD following specific stressful life events were assessed with the Impact of Event Scale (IES).^24^ All questionnaires demonstrated high internal consistency with Cronbach’s α of 0.79–0.94,^20^ 0.91,^21^ 0.71–0.85^23^ and 0.78–0.82.^24^ The first author (L.S.) administered all psychometric instruments using validated German translations.

### Experimental design

**Fig. 2:**
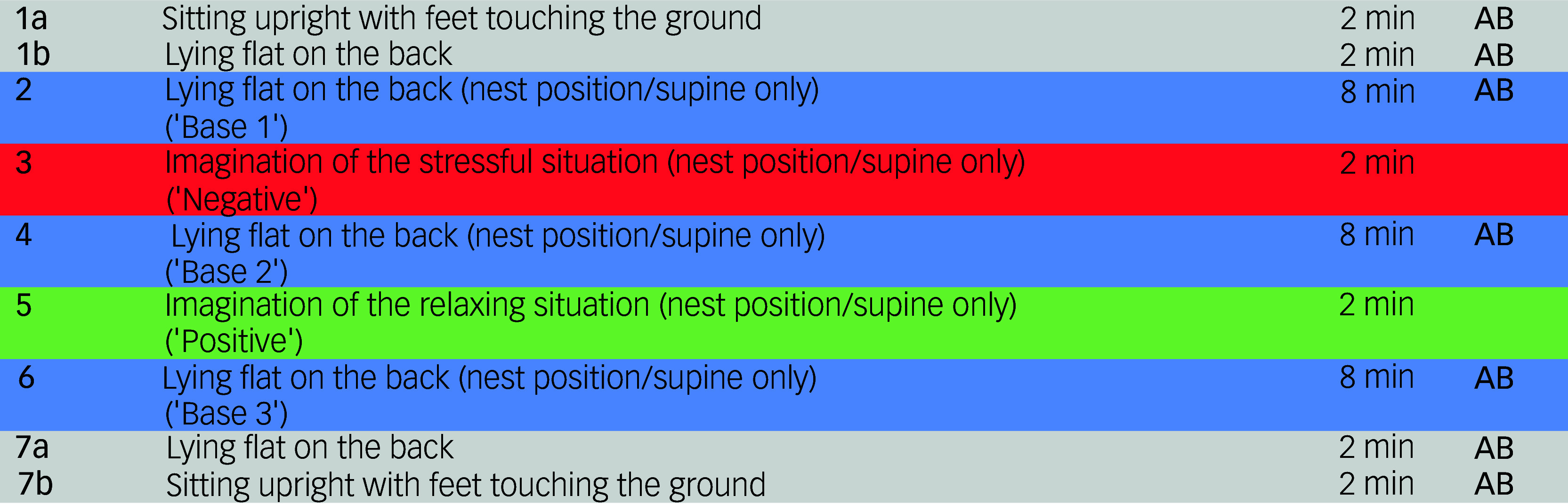
Course of the different measurement phases. The whole experiment consisted of two rounds of these different measurement phases. In one round of the experiment, the nest position was applied during steps 2 to 6, during the other round the participants lay in a neutral supine position only. AB, audiobook.

The experiment took place in a physiotherapy treatment room at the Department of Psychosomatic Medicine and Psychotherapy of the above-mentioned clinics from 9 June 2019 to 7 November 2021. It involved a measurement session lasting approximately 90 minutes, conducted by the first author (L.S.). The experimental process, including the different measurement phases, is depicted in Fig. 2.

Ahead of the experiment, participants were asked to define both a stressful and a comforting situation based on the Eye Movement Desensitization and Reprocessing (EMDR) manual^25^ and were instructed to use keywords to describe these situations, including the associated cognitions, bodily reactions and emotions.

The experimental procedure consisted of two rounds and two experimental conditions (nest position or supine only): Before the first round, patients were randomised 1:1 to the experimental conditions by the first author (L.S.). In the second round, they were assigned to the alternate condition based on the first-round allocation. For the healthy controls, the order of experimental conditions was matched to that of the corresponding patient counterpart. The main measurement phases consisted of three 8 min baseline phases (‘Base 1’, ‘Base 2’, ‘Base 3’) alternating with 2 min measurement periods, during which participants were instructed to silently visualise the predetermined stressful or comforting situation (‘Negative’/‘Positive’). During these measurement phases, participants were either surrounded by blankets and other soft material forming a ‘nest’ while lying on their back (Fig. 1; nest position) or were only lying in a neutral supine position (supine only). The experiment commenced and concluded with participants sitting on the physiotherapy treatment table with their feet touching the ground, and lying in a neutral supine position for two minutes each (Fig. 2). Each step of the measurement session was explained in detail, and the participants were encouraged to communicate any distress related to the experimental procedure immediately.

Except during the ‘Negative’ and ‘Positive’ measurement phases, participants listened to a neutral non-fiction audiobook of their choice. This was intended to maintain their attention and help them stay focused and engaged in the experiment, thereby minimising the risk of trauma-related symptoms such as spontaneous dissociation or intrusions during the baseline phases. The audiobook was selected from five options prior to the experiment and should elicit neither positive nor negative emotions. Further details on the available audiobooks and the assessment of the participants’ perception are provided in Supplementary Material 1 available at https://doi.org/10.1192/bjo.2025.10072.

### Measured parameters

#### Self-reported distress and comfort

We assessed the participants’ subjective level of distress using the Subjective Units of Distress (SUD) scale.^26^ To assess comfort, we collected Subjective Units of Comfort (SUC) similarly. To the best of our knowledge, this instrument has not been previously applied in a similar context. Following each measurement phase, participants were asked first to rate their current stress level on a scale from zero to ten, and then their current level of comfort with zero indicating minimal stress/comfort and ten signifying maximum stress/comfort.

#### Psychophysiology

Different psychophysiological parameters were continuously monitored through the Vrije Universiteit Ambulatory Monitoring System (VU-AMS), a wearable device designed to non-invasively record autonomic nervous system (ANS) activity using electro- and impedance cardiography.^27^

The inter-beat interval (IBI), measured in milliseconds (ms), indicates the time between two consecutive heartbeats. It can be converted into heart rate (HR) in beats per minute by dividing 60 000 by the IBI.^28^ The pre-ejection period (PEP) (in ms) reflects cardiac contractility and is considered a measure of sympathetic nervous system activity. Shorter PEP-values indicate a higher sympathetic tone.^29^ The root mean square of successive differences (RMSSD) (in ms) is calculated using the IBI, quantifies heart rate variability (HRV) and serves as an index of cardiac vagal control. An increase in RMSSD indicates a stronger parasympathetic drive.^30^ Due to the known skewed distribution of the RMSSD, we used the natural logarithm (ln)RMSSD in the present study.^31^

### Data analytic plan

Physiological data were processed using the VU Data Analysis and Management Software for Windows, version 5.0 (Vrije Universiteit Amsterdam, Amsterdam, The Netherlands; see https://vu-ams.nl/downloads/). We considered the main measurement phases (i.e., ‘Base 1’, ‘Base 2’, ‘Base 3’, ‘Negative’, ‘Positive’) and estimated the mean values of the psychophysiological parameters from the first minute of each phase to capture the immediate effects of the introduced experimental condition. While 5 min intervals are generally recommended, e. g. for HRV measurement, official guidelines allow for shorter durations when justified by the study design.^30^

All statistical calculations were performed using SPSS Statistics for Windows, version 27.0 (IBM, Armonk, NY, USA; see https://www.ibm.com/de-de/products/spss-statistics), with a predefined statistical threshold of *p* < 0.05 (two-tailed). The hypotheses were tested separately for the patients and the healthy controls, focusing on intra-individual differences between the nest position and supine only. Normally distributed metric variables were compared using paired-sample *t*-tests. Otherwise, we calculated Wilcoxon signed-rank tests. For nominal variables, *χ* ^2^-tests were applied. In order to estimate full model effects, linear mixed models (LMM) were applied with participants as random effects, and group allocation and experimental condition (nest position or supine only) as fixed effects. A factor for the sequence of the application of the nest position (first or in the second round after supine only) did not reveal any significant effect and was thus omitted from the LMM. Finally, we calculated effect sizes (Hedges’ *g*, due to a sample size of less than 20 in each group, with 0.2 = small effect, 0.5 = medium effect and 0.8 = large effect^32^).

## Results

### Sample characteristics

**Fig. 3:**
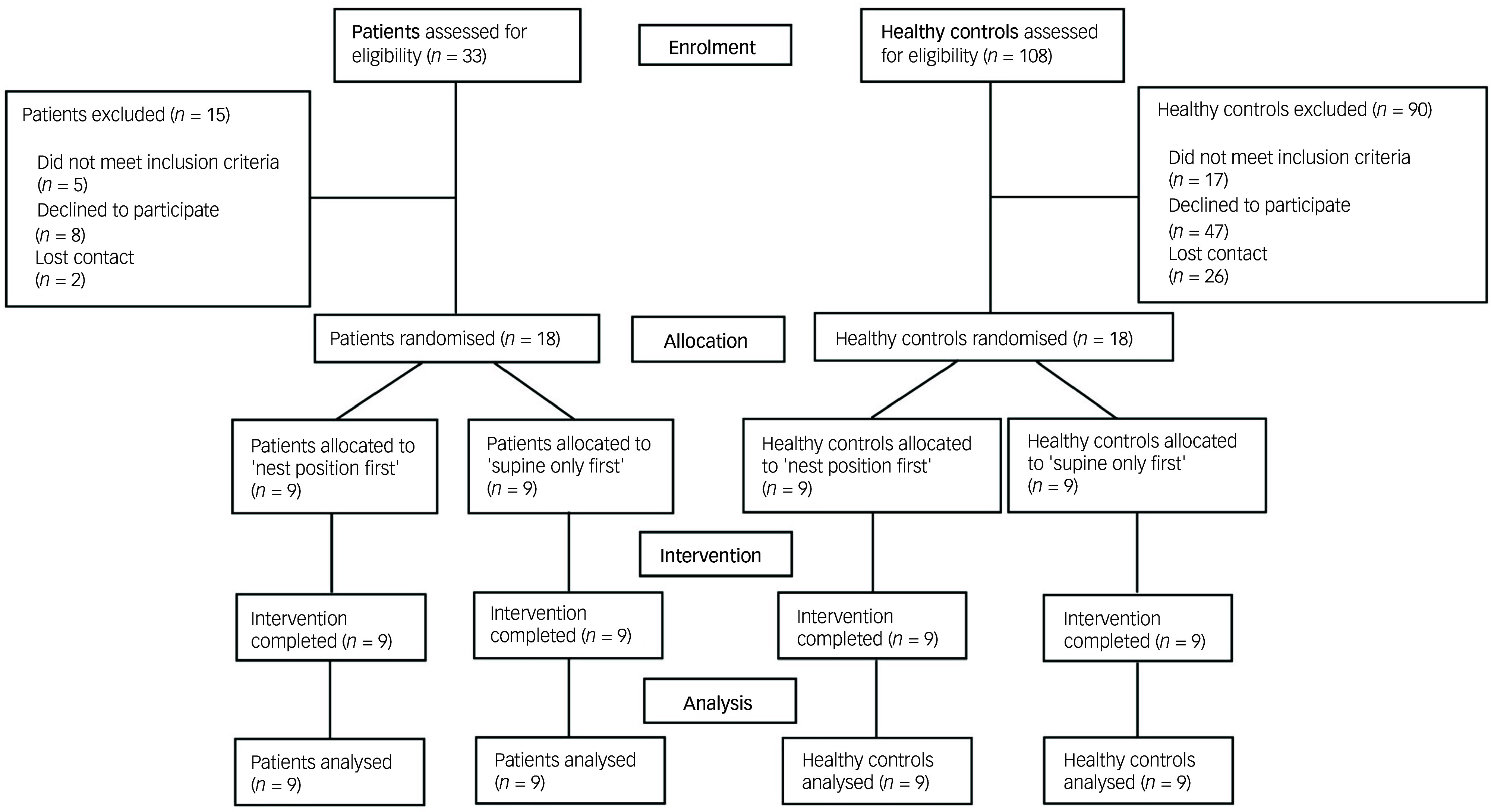
CONSORT flow diagrams.

The CONSORT flow charts in Fig. 3 outline the number of enrolled, randomised and analysed participants. The study ended after 18 female patients and 18 female healthy controls had successfully completed the measurement session. The sociodemographic and psychometric data are summarised in Table 1. The mean values for age and BMI were 42.6 (s.d. = 12.3) years and 27.0 (s.d. = 5.4) kg/m^2^ for the patients and 42.7 (s.d. = 12.5) years and 25.6 (s.d. = 5.0) kg/m^2^ for the healthy controls. There were no significant differences between the groups regarding gender (*p* = 1.0), age (*p* = 0.97) and BMI (*p* = 0.44). All healthy controls were employed, compared to 61.1% of the patients. Forty-four per cent of the patients and two-thirds of the healthy controls held a high school diploma. Most participants were single (55.6% of the patients vs. 61.1% of the healthy controls), with one-sixth of each group being married. The remainder were separated, divorced or widowed.

**Table 1:** Sample characteristics

Variable	Patients (*n* = 18)		Healthy controls (*n* = 18)	
	Mean	s.d.	Mean	s.d.
Age	42.56	12.28	42.72	12.54
Body mass index	26.96	5.38	25.61	5.01
Months in in-patient care				
Psychosomatic	5.14	5.07	–	–
Psychiatric	1.42	2.85	–	–
Mini-SCID-D				
Total score	13.33	1.75	0.22	0.43
Subscale amnesia	2.72	0.57	0.00	0.00
Subscale depersonalisation	3.00	0.00	0.11	0.32
Subscale derealisation	2.39	1.04	0.00	0.00
Subscale identity confusion	2.83	0.38	0.11	0.32
Subscale identity alteration	2.22	0.55	0.00	0.00
DES total score	38.03	17.14	4.15	3.94
IES				
Total score	47.44	20.12	–	–
Subscale intrusion	22.06	10.00	–	–
Subscale avoidance	25.39	11.83	–	–
CTQ				
Total score	15.44	4.08	5.78	0.62
Subscale physical abuse	2.69	1.26	1.04	1.00
Subscale emotional abuse	4.08	1.07	1.48	0.40
Subscale sexual abuse	2.43	1.24	1.03	0.14
Subscale physical neglect	2.44	0.88	1.06	0.11
Subscale emotional neglect	3.95	0.94	1.48	0.40
BSI Global Severity Index^a^	2.11	0.44	0.20	0.13

BSI, Brief Symptom Inventory (range: 0–4); CTQ, Childhood Trauma Questionnaire (range total score: 0–25; range subscales: 0–5); DES, Dissociative Experience Scale (range: 0–100%); IES, Impact of Event Scale (range total score: 0–75; range subscale intrusion: 0–35; range subscale avoidance: 0–40); Mini-SCID-D, short form of the Structured Clinical Interview for DSM-IV Dissociative Disorders (range total score: 0–15; range subscales: 0–3).

a Sum of all subscale scores divided by 53.

All patients were diagnosed with dissociative disorders (DDNOS Type 1) with an average score of 13.3 (s.d. = 1.8; range: 10–15) points in the Mini-SCID-D interview (range: 0–15) and met the criteria for PTSD according to the SCID-PTSD. Mini-SCID-D subscale scores (range: 0–3) indicated high depersonalisation (mean = 3.00; s.d. = 0.00) and moderate-high amnesia (mean = 2.72; s.d. = 0.57), derealisation (mean = 2.39; s.d. = 1.04), identity confusion (mean = 2.83; s.d. = 0.38) and identity alteration (mean = 2.22; s.d. = 0.55). Patients scored an average of 47.44 (s.d. = 20.12) points on the IES (range: 0–75), corresponding to a severe impact from stressful life events, and experienced both avoidance and intrusion with high intra-group variability. Comorbidities included affective (88.9%), personality (27.8%), eating (16.7%) and anxiety or obsessive–compulsive disorders (11.1%). Nearly half of the patients (44.4%) were taking antidepressants, 22.2% antipsychotics and 11.1% both types of medication. As to previous treatment, the patients had spent on average 5.1 (s.d. = 5.1) months in psychosomatic and 1.4 (s.d. = 2.9) months in psychiatric in-patient care. None of the healthy controls fulfilled the diagnostic criteria for PTSD or came close to the cut-off value in the Mini-SCID-D interview (mean = 0.20; s.d. = 0.40; range 0–1). The mean total score on the DES (range 0–100%) was 38.0% (s.d. = 17.1%) for the patients and 4.2% (s.d. = 3.9%) for the healthy controls, indicating significantly higher trait dissociation in the patients (*p* < 0.001). Patients also reported significantly higher levels of childhood traumatisation on the CTQ (*p* < 0.001) and exhibited significantly higher psychological distress on the BSI (*p* < 0.001).

### Self-reported distress and comfort

**Fig. 4:**
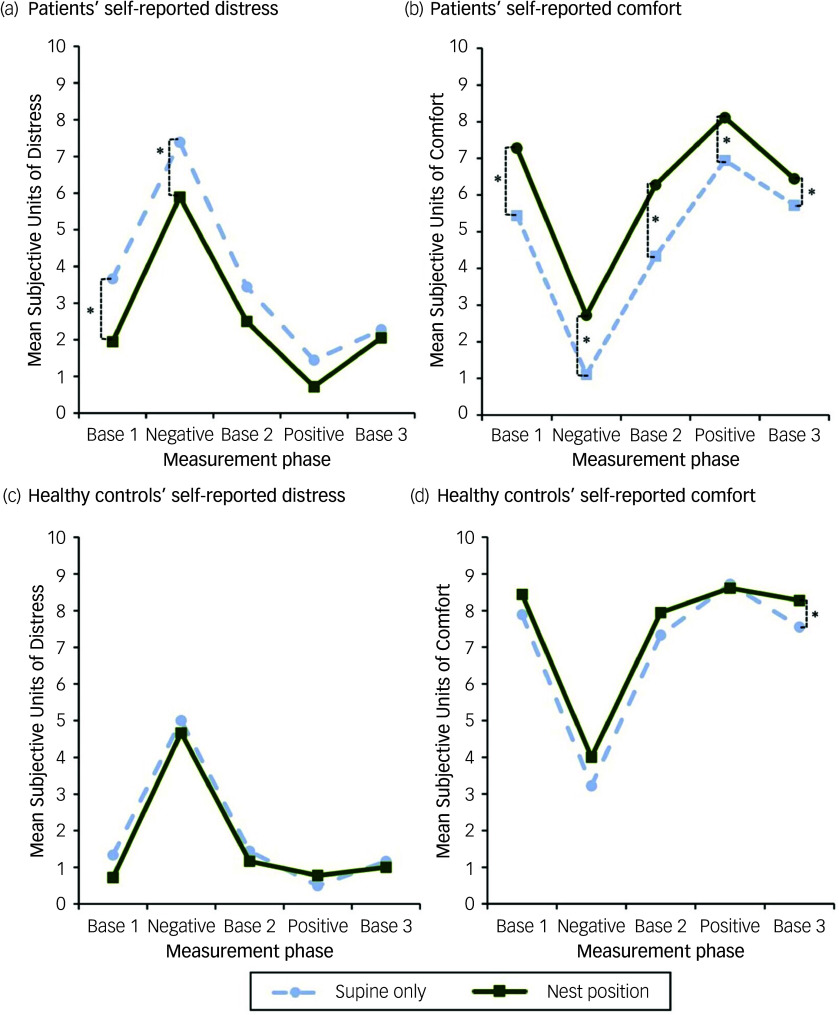
Courses of self-reported distress (Subjective Units of Distress, SUD) and self-reported comfort (Subjective Units of Comfort, SUC) for patients (a, b) and healthy controls (c, d). Base 1/2/3, first/second/third baseline condition; Negative/Positive, imagination of the predefined stressful/comforting situation. *p < 0.05.

The progressions of the SUD and the SUC over the course of the experiment are illustrated in Fig. 4, while Table 2 shows the means, s.d. and within-group differences. The results of the LMM are presented in more detail in Supplementary Material 2.

**Table 2:** Self-reported distress and comfort for patients and healthy controls

Variable	Patients (*n* = 18)							Healthy controls (*n* = 18)						
	Supine only		Nest position		*p* ^a^	*p*_adjust_^b^	Hedges’ *g*	Supine only		Nest position		*p* ^a^	*p*_adjust_^b^	Hedges’ *g*
	Mean	s.d.	Mean	s.d.				Mean	s.d.	Mean	s.d.			
SUD														
Base 1	3.67	2.20	1.94	1.43	**0.002**	**0.020**	−0.91	1.33	1.81	0.72	1.64	0.101	–	–
Negative	7.39	2.06	5.89	2.03	**0.004**	**0.029**	−0.72	5.00	1.33	4.67	1.85	0.392	–	–
Base 2	3.44	2.06	2.50	2.64	0.206	–	–	1.44	1.58	1.17	1.89	0.248	–	–
Positive	1.44	1.42	0.72	1.36	0.063	–	–	0.50	0.86	0.78	1.77	0.596	–	–
Base 3	2.28	2.02	2.06	2.18	0.419	–	–	1.17	1.42	1.00	1.40	0.549	–	–
SUC														
Base 1	5.44	2.55	7.28	1.27	**0.003**	**0.026**	0.89	7.89	2.25	8.44	2.23	0.077	–	–
Negative	1.11	1.97	2.72	2.22	**0.01**	**0.043**	0.75	3.22	2.56	4.00	2.95	0.084	–	–
Base 2	4.33	2.30	6.28	2.54	**0.015**	**0.043**	0.78	7.33	2.06	7.94	2.36	0.144	–	–
Positive	6.94	2.39	8.11	2.14	**0.03**	**0.043**	0.50	8.72	1.36	8.61	1.65	0.917	–	–
Base 3	5.72	1.90	6.44	2.23	**0.009**	0.051	0.34	7.56	2.33	8.28	2.19	**0.026**	0.26	0.31

Base 1/2/3, first/second/third baseline condition; Negative/Positive, imagination of the predefined stressful/comforting situation; SUC, Subjective Units of Comfort (range: 0–10); SUD, Subjective Units of Distress (range: 0–10).

a Within-group comparison between experimental conditions (nest position *v*. supine only).

b Adjusted *p*-values after Holm–Bonferroni correction.

Results in bold are statistically significant (*p* < 0.05).

#### Subjective units of distress

Regarding the SUD, patients reported lower distress levels in the nest position during all analysed measurement phases, with statistically significant differences observed for ‘Base 1’ (*p* = 0.002) and ‘Negative’ (*p* = 0.004). In both cases, effect sizes were large (*g* = –0.91; *g* = –0.72). Healthy controls reported only a little distress during all conditions apart from ‘Negative’. They generally exhibited a small numerical decrease in SUD while in the nest position, which did not reach statistical significance. LMM detected a statistically significant effect of both the group allocation and the experimental condition (nest position or supine only) on SUD during all baseline phases (both *p* < 0.001) and ‘Negative’ (*p* = 0.001 and *p* = 0.017). There was no significant group × condition interaction. None of the participants experienced an immediate aversive reaction to the nest position, discontinued the measurement session or required a psychological intervention afterwards. No physical harm was reported or observed.

#### Subjective units of comfort

Correspondingly, patients reported significantly higher levels of comfort during the nest position as compared with supine only in all three baseline phases (*p* = 0.003; *p* = 0.015; *p* = 0.009) as well as during ‘Negative’ (*p* = 0.01) and ‘Positive’ (*p* = 0.03) phases. Effect sizes were large for ‘Base 1’ (*g* = 0.89), ‘Base 2’ (*g* = 0.78) and ‘Negative’ (*g* = 0.75), while they were medium for ‘Positive’ (*g* = 0.50) and small for ‘Base 3’ (*g* = 0.34). In the healthy controls, a statistically significant difference was only observed during ‘Base 3’ with a small effect size (*p* = 0.026; *g* = 0.31). LMM indicated a statistically significant effect of both the group allocation and the experimental condition during all baseline phases (both *p* < 0.001) and ‘Negative’ (*p* = 0.029 and *p* = 0.040). For ‘Negative’, there was a significant group×condition interaction (*p* = 0.029) pointing towards more pronounced effects in the healthy controls.

### Psychophysiological parameters

**Fig. 5:**
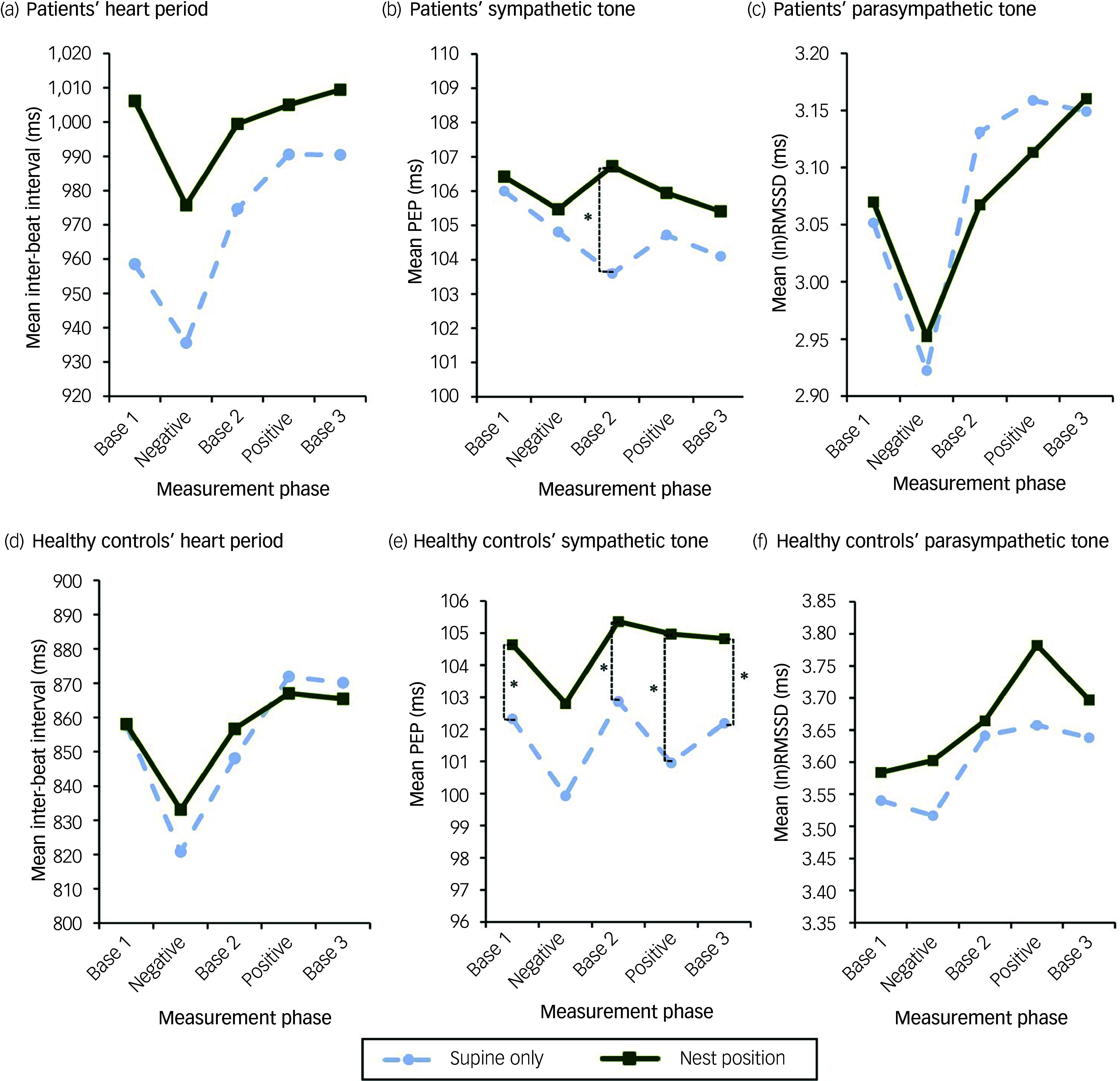
Courses of heart period (inter-beat interval, IBI), sympathetic tone (pre-ejection period, PEP) and parasympathetic tone (natural logarithm of the root mean square of successive differences, [ln]RMSSD) for patients (a, b, c) and healthy controls (d, e, f). Base 1/2/3, baseline conditions; Negative/Positive, imagination of the predefined stressful/comforting situation. *p < 0.05.

Figure 5 depicts the progression of the IBI, the PEP and the (ln)RMSSD during the experiment; the means, s.d. and within-group differences are presented in Table 3. The results of the LMM are presented in more detail in Supplementary Material 3.

**Table 3:** Psychophysiological parameters for patients and healthy controls

Variable	Patients (*n* = 18)							Healthy controls (*n* = 18)						
	Supine only		Nest position		*p* ^a^	*p*_adjust_^b^	Hedges’ *g*	Supine only		Nest position		*p* ^a^	*p*_adjust_^b^	Hedges’ *g*
	Mean	s.d.	Mean	s.d.				Mean	s.d.	Mean	s.d.			
IBI (ms)														
Base 1	857.71	111.39	858.13	122.47	0.979	–	–	958.54	145.97	1006.21	155.33	0.057	–	–
Negative	820.82	120.55	833.13	126.54	0.526	–	–	935.62	147.81	975.75	149.90	0.095	–	–
Base 2	848.142	116.63	856.68	117.97	0.685	–	–	974.69	127.25	999.46	147.12	0.249	–	–
Positive	871.93	118.59	867.02	107.86	0.781	–	–	990.58	139.63	1005.03	150.05	0.409	–	–
Base 3	870.10	107.86	865.42	110.14	0.815	–	–	990.41	151.85	1009.39	151.02	0.269	–	–
PEP (ms)														
Base 1	106.00	17.91	106.43	15.81	0.793	–	–	102.33	7.40	104.64	7.39	**0.045**	0.169	0.31
Negative	104.81	16.35	105.47	14.38	0.602	–	–	99.94	8.02	102.81	8.06	0.090	–	–
Base 2	103.60	16.40	106.73	15.34	**0.004**	0.060	0.19	102.87	9.08	105.35	6.59	**0.037**	0.278	0.31
Positive	104.72	16.60	105.95	15.74	0.152	–	–	100.96	8.18	104.97	7.27	**0.004**	0.060	0.51
Base 3	104.10	16.51	105.41	14.53	0.132	–	–	102.19	7.74	104.83	6.44	**0.023**	0.259	0.36
(ln)RMSSD														
Base 1	3.05	0.95	3.07	0.85	0.841	–	–	3.54	0.54	3.58	0.54	0.784	–	–
Negative	2.92	0.92	2.95	0.88	0.753	–	–	3.52	0.47	3.60	0.49	0.532	–	–
Base 2	3.13	0.89	3.07	0.87	0.457	–	–	3.64	0.57	3.66	0.47	0.851	–	–
Positive	3.16	0.81	3.11	0.78	0.626	–	–	3.66	0.48	3.78	0.58	0.177	–	–
Base 3	3.15	0.91	3.16	0.83	0.923	–	–	3.64	0.64	3.70	0.54	0.616	–	–

Base 1/2/3, first/second/third baseline condition; Negative/Positive, imagination of the predefined stressful/comforting situation; IBI, inter-beat interval; (ln)RMSSD, natural logarithm of the root mean square of successive differences; PEP, pre-ejection period.

a Within-group comparison between experimental conditions (nest position *v*. supine only).

b Adjusted *p*-values after Holm–Bonferroni correction.

Results in bold are statistically significant (*p* < 0.05).

In general, patients exhibited only small and non-significant effects for those parameters, whilst in the healthy controls more favourable changes could be observed for PEP and tendentially for IBI. Except for ‘Positive’ and ‘Base 3’, IBI was numerically but not significantly higher when the patients were lying in the nest position, indicating a lower heart rate. Similarly, we observed numerically higher IBI in the healthy controls during the nest position. While PEP was higher for the patients during the nest position, suggesting a lower sympathetic tone, the difference reached statistical significance only for ‘Base 2’, with a small effect size (*p* = 0.004; *g* = 0.19). In the healthy controls, PEP was significantly higher when the nest position was applied except during ‘Negative’ (‘Base 1’: *p* = 0.045; ‘Base 2’: *p* = 0.037; ‘Base 3’: *p* = 0.023; ‘Positive’: *p* = 0.004). Effect sizes ranged from small to medium (‘Base 1’ and ‘Base 2’: *g* = 0.31; ‘Base 3’: *g* = 0.36; ‘Positive’: *g* = 0.51). For both the patients and the healthy controls there were no significant differences in (ln)RMSSD, and thus parasympathetic tone, between the experimental conditions.

LMM detected a statistically significant effect of the group allocation during all three baseline phases on IBI (*p* < 0.001) and (ln)RMSSD (*p* < 0.001) but not on PEP. However, there was a statistically significant effect of the experimental condition on PEP (*p* = 0.020) indicating a more pronounced effect in the patients. For ‘Negative’, LMM showed a statistically significant effect of the group allocation on IBI (*p* = 0.010) and (ln)RMSSD (*p* = 0.011) but not on PEP. There was no significant group × condition interaction in any of these cases.

## Discussion

This study investigated the effects of the nest position, a physiotherapeutic intervention designed to promote safety and self-soothing, on adult female patients with dissociative disorders as well as healthy controls. Participants experienced less distress and higher comfort during the nest position. Although we did not observe substantial changes in the parasympathetic tone, both the heart rate and sympathetic tone decreased.

### Self-reported distress and comfort

During the nest position, we observed a notable reduction in self-reported distress for both the patients and the healthy controls. Patients reported significantly higher comfort in all measurement phases compared to a neutral supine position only, while healthy controls showed non-significant increases. LMM detected a significant positive overall effect of the nest position on both self-reported stress and comfort.

To the best of our knowledge, no studies have so far investigated the effects of physiotherapeutic interventions on distress levels in patients with dissociative disorder. However, our findings align with studies by Price^33^ and Classen et al,^34^ which demonstrated that body-therapy interventions can reduce post-traumatic and dissociative symptoms while improving self-soothing skills in women with a history of childhood trauma.

The significant increase in comfort during the nest position suggests that our intervention addresses important aspects of treatment in DD: providing safety and strengthening the ability to self-soothe.^9^ There are already therapy programmes for patients with PTSD that aim to target both these concepts, such as CARESS^35^ and ‘Seeking Safety’.^36^ For patients with dissociative disorders, participation in an online psychoeducational and skill-building programme was associated with symptom reduction, improved emotion regulation and increased safety.^37,38^ The nest position might help bridge the gap in well-researched acute interventions that focus on both safety and self-soothing, while also considering the bodily experience – especially, as the nest position can be applied easily by the patients themselves at home or in other convenient locations.

Some concepts and techniques resembling the nest position have generally proven effective in reducing distress and increasing comfort, when applied in other target groups. For example, swaddling with ribbons, folded blankets or sheets calms infants, promotes their sleep and reduces both pain and crying.^39^ In the context of the Newborn Individualized Developmental Care and Assessment Program (NIDCAP), postures that help align the adducted and flexed extremities to the midline have been associated with positive effects on self-regulation and feelings of security in premature babies.^40^ The nest position might also serve as a sensory modulation intervention as it incorporates elements of deep-touch pressure. This is a form of tactile sensory input typically elicited by firm pressure, such as in weighted blankets, which might elicit feelings of being held, safe and comforted.^41^

Even during more stressful parts of the measurement session, the patients reported feeling safe and comforted, which allowed them to complete the experiment. None experienced an aversive reaction to the first author (L.S.) applying the nest position, discontinued the experiment or required a psychological intervention afterwards. This might be an indicator for the high acceptability of the nest position. After the experiment, some participants found the confinement of the nest position unfamiliar and, in some cases, unpleasant, as it prevented them from escaping the imagined stressful situation and its associated emotions. This may explain the higher-than-expected SUD scores during certain measurement phases. As Brand et al^42^ note, highly traumatised patients with dissociative disorders may initially experience distress when feeling relaxed or safe, having previously relied on unsafe behaviours for emotion regulation. Clinicians must recognise that feelings of safety and comfort can themselves be triggering and distressing, especially early on in the treatment.

Comfort is a subjective and context-dependent construct commonly associated with sensations of satisfaction, calmness, relaxation and relief. Current measures of comfort are rare and predominantly focus on freedom from physical pain, with no established gold standard existing for its assessment.^43^ Until now, limited focus has been directed to research into the role and measurement of feelings of comfort in patients with trauma-related disorders, particularly dissociative disorders. Measures of psychological safety have traditionally been limited to specific contexts, such as team dynamics or childhood memories, as indicated by Morton et al.^44^ Their recently developed Neuroception of Psychological Safety Scale had not yet been available during our data collection. We suggest that the SUC used in our study may be appropriate and practical for evaluating psychological comfort, and potentially even safety. However, analogous to the SUC, Subjective Units of Safety (SUS) may represent an even more adequate psychometric scale for assessing feelings of safety, which should be explored in further studies.

### Psychophysiology

Contrary to our hypotheses, there was no significant increase in the parasympathetic tone in either the patients or the healthy controls during the nest position. However, both groups exhibited a numerically lower heart rate and significantly reduced sympathetic tone. LMM detected a significant effect of the nest position on PEP during the baseline phases, with a more pronounced impact in the patient group.

Previous research on psychophysiological parameters in dissociative disorders has primarily focused on stress reactions. To our knowledge, the modulation of psychophysiological parameters during a comforting intervention has not been explored in patients with dissociative disorders. Studies on interventions resembling the nest position have shown that bedding with soft material can have positive effects on the ANS: weighted blankets and vests, which apply deep-touch pressure, reduce the sympathetic tone in healthy adults.^45^ The calming effect of positioning techniques for infants has also been attributed to an increase in the parasympathetic tone.^46^

There remains disagreement concerning the exact psychophysiology of dissociative disorders. Recent reviews have reported no clear trends in various psychophysiological parameters in trauma-related dissociation.^47,48^ Therefore, it is plausible that changes in the ANS during states of comfort and safety may also exhibit various patterns.

In the present study, there was a notable discrepancy between the significant reduction in self-reported stress, the significant increase in comfort levels and the modest changes in the psychophysiological variables among the patients. Interestingly, this pattern was not seen in the healthy controls, where the changes in the ANS activity were partly even more pronounced than the shifts in self-reported stress and comfort. This finding may indicate a blunted ANS reactivity in patients with dissociative disorders, aligning with studies on the dissociative subtype of PTSD. According to Orr et al,^49^ approximately one-quarter to one-third of clinically confirmed PTSD patients do not exhibit psychophysiological hyperreactivity. Other studies indicate that these patients are precisely those experiencing dissociative symptoms (e.g. ^31^). Similarly, autonomic hypoarousal is observed in neutral identity states compared to trauma-related identity states in individuals with dissociative identity disorder.^50,51^

Additionally, demand characteristics may explain the disparity between SUD scores and the small differences in the psychophysiological parameters. Patients might have reported lower distress and higher comfort than they experienced to conform as ‘good’ subjects.^52^ However, during recruitment we withheld details about the intervention, hypotheses and the exact course of the experiment. The nest position was only described as an innovative physiotherapeutic intervention, without revealing its name or specific method, to ensure that participants approached the experiment as unbiased as possible.

Our results also point towards a pathologically rigid ANS response to different emotional stimuli in patients with dissociative disorders. According to Thayer et al,^53^ this rigidity can be interpreted as impaired emotion regulation. Additionally, a flexible vagal tone appears to be associated with feeling safe and a greater ability to self-soothe.^54^ The changes in the psychophysiological parameters may not have been as significant as anticipated because all participants experienced the intervention for the first time and a single session might have been insufficient to substantially influence the ANS and such complex functions as emotion regulation.

### Limitations and strengths

A key limitation of the present study is the small sample size (*n* = 36). Male participants were not included, and we did not standardise at which point in the course of the psychotherapeutic treatment the patients participated in the study. However, a strength of our study is the thorough assessment of dissociative disorders and PTSD using structured clinical interviews. Nonetheless, we did not evaluate detachment and compartmentalisation symptoms, even though the DES is suitable for such assessments.^55^

When interpreting the significance of our findings, it is important to acknowledge the large number of intra-group comparisons conducted. After the application of a Holm–Bonferroni correction, several results no longer reach statistical significance, including the SUC in ‘Base 3’ (Table 2) and all previously statistically significant differences in the psychophysiological parameters (Table 3).

We did not conduct correlation analyses between self-reported distress/comfort and the psychophysiological parameters, although they might have provided additional insights. Given the small sample size and multiple comparisons involved in the present study, such elaborate calculations would be challenging. Additionally, the decoupling of the subjective experiences and psychophysiological responses in individuals with dissociative disorders and PTSD^56^ further complicates interpretation.

It is possible that the chosen 1 min intervals for analysing the psychophysiological parameters may not have been optimal, as longer recording periods might have allowed for the detection of long-term trends. However, we selected this interval length to capture changes directly associated with the intervention, which may wear off quickly.^57^ Moreover, for assessing ANS activity and monitoring mental stress, short-term analyses of HR within 10 s and RMSSD within 30 s are considered to be as reliable as the guideline-recommended 5 min intervals.^58^

Other limitations might have arisen from the randomisation conducted by the first author (L.S.) and her presence throughout the experiment, as well as the use of individually tailored components, such as the selected audiobook, to minimise additional stress for the patients. Furthermore, we were unable to objectify the depth of imagination of the stressful and comforting situation. We also did not systematically determine whether patients alternated between neutral and trauma-related identity states. This could have affected the interpretation of psychophysiological data, given that autonomic hypoarousal has been observed in neutral identity states.^50,51^ However, no participant showed overt signs of shifts between identity states (e. g. changes in speech or body language) or reported such experiences during the experiment.

Lastly, relying solely on the SUD and SUC may not have been entirely suitable for confirming our hypotheses. As previously mentioned, suitable psychometric measures of psychological comfort and safety were not available during our data collection and in future studies, the perception of safety and self-soothing should be explicitly assessed, e.g. by using SUS.

### Clinical implications

The nest position emerges as a potentially promising intervention that might be able to enhance existing effective treatment regimens in patients with dissociative disorders. By possibly providing a physical sense of safety and comfort as well as facilitating self-soothing, it may effectively reduce distress.

Additionally, the present study addresses the significant research gap in the field of dissociative disorders and physiotherapy within inpatient mental health care. Given the substantial burden of dissociative disorders and the high prevalence of dissociative symptoms in various mental disorders, suitable and personalised treatment interventions need to be developed. Patients might benefit from approaches that emphasise the bodily experience of emotions, warranting further research into these treatment modalities.

Lastly, our findings contribute to a better understanding of the importance of self-soothing and safety in highly dissociative patients. Developing valid and reliable measures of psychological comfort and safety is necessary, although the new SUC used in our study may serve as a practical tool in this regard. According to the current treatment guideline of dissociative disorders, ‘without the attention to the myriad of safety problems […] little will be accomplished in the treatment’.^59^ Rather than solely focusing on reducing arousal, it is recommended to foster feelings of safety and assist patients in self-soothing.

## Supporting information

Stief et al. supplementary material Stief et al. supplementary material

## Data Availability

The data that support the findings of this study are not publicly available due to privacy restrictions. The data are available from the corresponding author, E.S., upon reasonable request.

## Supplementary material

The supplementary material is available online at https://doi.org/10.1192/bjo.2025.10072
