# Supplementary material for: Safe and soothed: randomised clinical pilot study on the subjective and psychophysiological impact of a new physiotherapeutic intervention in patients with dissociative disorders
Source: BJPsych Open. 2025 Sep 10;11(5):e198. doi: 10.1192/bjo.2025.10072 (PMC12451716; doi:10.1192/bjo.2025.10072)
Supplement: Stief et al. supplementary material [file S2056472425100720sup001.docx]

**Supplementary Materials**

**Supplementary Material 1. Audiobook selection and assessment of perception**

Before the experiment, participants were given the choice of five audiobooks in German. The available options, listed alphabetically, were:

1. Forester V. Lessing und Moses Mendelssohn – Geschichte einer Freundschaft [Lessing and Moses Mendelssohn – History of a Friendship]. Kopenhagen: SAGA Egmont; 2017.
2. Fündling J. Die Welt Homers [The World of Homer]. Kopenhagen: SAGA Egmont; 2018.
3. Gleba G. Klöster und Orden im Mittelalter [Monasteries and Religious Orders in the Middle Ages]. Kopenhagen: SAGA Egmont; 2018.
4. Kondo M. Magic Cleaning (Wie richtiges Aufräumen Ihr Leben verändert) [Magic Cleaning (How Tidying up Can Change Your Life)]. Berlin: Argon Balance; 2013.
5. Pape G. Gebrauchsanweisung fürs Gärtnern [Instructions for Gardening]. Bochum: tacheles!; 2018.

Among the patients, the most frequently chosen audiobook was the fourth option (Magic Cleaning; 38,9%), followed by the second with 22,2% (Homer). The first (Lessing) and third (Middle Ages) audiobooks were each selected by 16.7%, while the fifth option (Gardening) was preferred by a single patient (5.6%). Among the healthy controls, Magic Cleaning was again the most popular choice (27.8%), followed by the second and third audiobook (Homer and Middle Ages; 22.2% each). The fifth audiobook (Gardening) was selected by 16.7%, while the first audiobook (Lessing) was chosen by 11.1%.

No standardized assessment of the perception of the audiobooks was conducted. However, before the start of the experiment, we ensured by asking that the chosen option did not elicit strong emotional responses. Most participants described their selection as primarily inducing boredom.

**Supplementary Material 2. Results of the linear mixed models for subjective distress and comfort**

| Fixed effects | **Baseline phases** | | | | | | | |
| --- | --- | --- | --- | --- | --- | --- | --- | --- |
|  | **SUD** | | | | **SUC** | | | |
|  | ***Estimate*** | ***SE*** | ***F*** | ***p*** | ***Estimate*** | ***SE*** | ***F*** | ***p*** |
| **Constant term** |  |  | 158.87 | **< 0.001** |  |  | 1720.82 | **< 0.001** |
| **Group allocation** |  |  | 44.02 | **< 0.001** |  |  | 25.22 | **< 0.001** |
| **Experimental condition** |  |  | 45.51 | **< 0.001** |  |  | 38.57 | **< 0.001** |
| Patients: Nest position | 1.55 | 0.17 |  |  | 7.08 | 0.27 |  |  |
| Patients: Supine only | 3.87 | 0.36 |  |  | 5.45 | 0.41 |  |  |
| Healthy controls: Nest position | 1.00 | 0.17 |  |  | 9.30 | 0.27 |  |  |
| Healthy controls: Supine only | 1.59 | 0.36 |  |  | 6.67 | 0.41 |  |  |
| **Group×condition interaction** |  |  | 2.15 | 0.147 |  |  | 2.12 | 0.149 |
|  | **Negative** | | | | | | | |
|  | **SUD** | | | | **SUC** | | | |
|  | ***Estimate*** | ***SE*** | ***F*** | ***p*** | ***Estimate*** | ***SE*** | ***F*** | ***p*** |
| **Constant term** |  |  | 572.45 | **< 0.001** |  |  | 58.31 | **< 0.001** |
| **Group allocation** |  |  | 13.49 | **0.001** |  |  | 5.25 | **0.029** |
| **Experimental condition** |  |  | 6.55 | **0.017** |  |  | 4.57 | **0.040** |
| Patients: Nest position | 6.00 | 0.61 |  |  | 1.89 | 0.76 |  |  |
| Patients: Supine only | 7.78 | 0.35 |  |  | 2.00 | 0.70 |  |  |
| Healthy controls: Nest position | 4.67 | 0.61 |  |  | 5.22 | 0.76 |  |  |
| Healthy controls: Supine only | 5.44 | 0.35 |  |  | 2.00 | 0.70 |  |  |
| **Group×condition interaction** |  |  | 1.00 | 0.326 |  |  | 5.25 | **0.029** |

*Note.* Results in bold are statistically significant (*p* < 0.05). Negative, imagination of the predefined stressful situation; SE, standard error; SUC, Subjective Units of Comfort (range: 0–10); SUD, Subjective Units of Distress (range: 0–10)

**Supplementary Material 3. Results of the linear mixed models for psychophysiological parameters**

| Fixed effects | **Baseline phases** | | | | | | | | | | | |
| --- | --- | --- | --- | --- | --- | --- | --- | --- | --- | --- | --- | --- |
|  | **IBI** | | | | **PEP** | | | | **lnRMSSD** | | | |
|  | ***Estimate*** | ***SE*** | ***F*** | ***p*** | ***Estimate*** | ***SE*** | ***F*** | ***p*** | ***Estimate*** | ***SE*** | ***F*** | ***p*** |
| **Constant term** |  |  | 5087.96 | **< 0.001** |  |  | 8120.13 | **< 0.001** |  |  | 2140.40 | **< 0.001** |
| **Group allocation** |  |  | 28.53 | **< 0.001** |  |  | 0.34 | 0.563 |  |  | 14.27 | **< 0.001** |
| **Experimental condition** |  |  | 0.03 | 0.868 |  |  | 5.62 | **0.020** |  |  | 1.38 | 0.242 |
| Patients: Nest position | 825.08 | 27.59 |  |  | 109.67 | 2.58 |  |  | 3.22 | 0.16 |  |  |
| Patients: Supine only | 834.49 | 22.44 |  |  | 100.67 | 2.02 |  |  | 2.79 | 0.13 |  |  |
| Healthy controls: Nest position | 964.62 | 27.59 |  |  | 104.82 | 2.58 |  |  | 3.49 | 0.16 |  |  |
| Healthy controls: Supine only | 963.59 | 22.44 |  |  | 102.83 | 2.02 |  |  | 3.58 | 0.13 |  |  |
| **Group×condition** |  |  | 0.04 | 0.836 |  |  | 2.28 | 0.134 |  |  | 3.43 | 0.067 |
|  | **Negative** | | | | | | | | | | | |
|  | **IBI** | | | | **PEP** | | | | **lnRMSSD** | | | |
|  | ***Estimate*** | ***SE*** | ***F*** | ***p*** | ***Estimate*** | ***SE*** | ***F*** | ***p*** | ***Estimate*** | ***SE*** | ***F*** | ***p*** |
| **Constant term** |  |  | 1350.99 | **< 0.001** |  |  | 2443.77 | **< 0.001** |  |  | 665.51 | **< 0.001** |
| **Group allocation** |  |  | 7.53 | **0.010** |  |  | 1.09 | 0.304 |  |  | 7.47 | **0.011** |
| **Experimental condition** |  |  | 0.00 | 0.967 |  |  | 1.81 | 0.188 |  |  | 1.65 | 0.209 |
| Patients: Nest position | 785.24 | 51.57 |  |  | 109.11 | 4.35 |  |  | 3.07 | 0.29 |  |  |
| Patients: Supine only | 804.82 | 41.37 |  |  | 101.75 | 3.99 |  |  | 2.58 | 0.19 |  |  |
| Healthy controls: Nest position | 935.05 | 51.57 |  |  | 103.02 | 4.35 |  |  | 3.57 | 0.29 |  |  |
| Healthy controls: Supine only | 911.53 | 41.37 |  |  | 99.12 | 3.99 |  |  | 3.42 | 0.19 |  |  |
| **Group×condition** |  |  | 0.21 | 0.648 |  |  | 0.17 | 0.681 |  |  | 0.49 | 0.489 |

*Note.* Results in bold are statistically significant (*p* < 0.05). Negative, imagination of the predefined stressful situation; IBI, inter-beat interval; (ln)RMSSD, natural logarithm of the root mean square of successive differences; PEP, pre-ejection period; SE, standard error
